# A PB1-K577E Mutation in H9N2 Influenza Virus Increases Polymerase Activity and Pathogenicity in Mice

**DOI:** 10.3390/v10110653

**Published:** 2018-11-19

**Authors:** Haruhiko Kamiki, Hiromichi Matsugo, Tomoya Kobayashi, Hiroho Ishida, Akiko Takenaka-Uema, Shin Murakami, Taisuke Horimoto

**Affiliations:** Department of Veterinary Microbiology, Graduate School of Agricultural and Life Sciences, The University of Tokyo, 1-1-1 Yayoi, Bunkyo-ku, Tokyo 113-8657, Japan; kamiki-haruhiko295@g.ecc.u-tokyo.ac.jp (H.K.); matsugo-hiromichi328@g.ecc.u-tokyo.ac.jp (H.M.); kobatomo49@g.ecc.u-tokyo.ac.jp (T.K.); ishida-hiroho417@g.ecc.u-tokyo.ac.jp (H.I.); atakiko@mail.ecc.u-tokyo.ac.jp (A.T.-U.); amurakam@mail.ecc.u-tokyo.ac.jp (S.M.)

**Keywords:** influenza, H9N2, mouse adaptation, polymerase, PB1

## Abstract

H9N2 avian influenza viruses are present in poultry worldwide. These viruses are considered to have pandemic potential, because recent isolates can recognize human-type receptor and several sporadic human infections have been reported. In this study, we aimed to identify mutations related to mammalian adaptation of H9N2 influenza virus. We found that mouse-adapted viruses had several mutations in hemagglutinin (HA), PB2, PA, and PB1. Among the detected mutations, PB1-K577E was a novel mutation that had not been previously reported to involve mammalian adaptation. A recombinant H9N2 virus bearing only the PB1-K577E mutation showed enhanced pathogenicity in mice, with increased virus titers in nasal turbinates compared to that in mice infected with the wild-type virus. In addition, the PB1-K577E mutation increased virus polymerase activity in human cell culture at a lower temperature. These data suggest that the PB1-K577E mutation is a novel pathogenicity determinant of H9N2 virus in mice and could be a signature for mammalian adaptation.

## 1. Introduction

The natural reservoirs of influenza A viruses are aquatic birds; however, these viruses are occasionally transmitted to fowl or mammals. Although most cases are transient, in rare cases, an influenza A virus can cause a pandemic by jumping species barriers and establishing a new lineage in a new host. To date, the H1, H2, H3, H5, H6, H7, H9, and H10 subtypes of influenza A viruses have been shown to infect humans [1,2,3,4,5]. Among these subtypes, only H1, H2, and H3 have become endemic in humans as seasonal flu. Although previous studies have shown that hemagglutinin (HA) and viral polymerase are crucial determinants for adaptation to mammals [6,7], the detailed mechanisms are not fully understood.

H9N2 avian influenza virus was first isolated from a turkey in the United States in 1966 [8], and since then the virus has spread throughout the world. H9N2 virus infection of poultry causes weight loss and decreased egg production, resulting in significant economic losses [9,10]. In addition to infection with the H9N2 virus itself, some of the H9N2 virus genes were transferred to highly pathogenic H5N1 and H7N9 viruses; and the first human infections with these viruses were reported in Hong Kong in 1997 and China in 2013, respectively [11,12,13].

In 1999, the first human H9N2 avian influenza virus infection was reported in Hong Kong [1]. Human cases of H9N2 infection were subsequently reported in China, Bangladesh, and Egypt [1,14,15]. Furthermore, serological evidence of human infection has also been reported in other countries in Asia, the Middle East, Africa, and North America [16], raising public health concern that the H9N2 virus may become a pandemic virus in humans. In addition to these cases of human infection, pigs [17], dogs [18], cats [19], and some primates [20] have also been naturally infected by H9N2 viruses. Under experimental conditions, mice could be infected with H9N2 viruses without prior adaptation [21], and some strains caused lethal infection [9,21]. The molecular basis of cross-species infection has been examined in the most recent H9N2 viruses, and these viruses harbor a leucine at position 226 of HA [22], which is involved in the recognition of human-type receptors [23] and direct contact transmission of H9N2 viruses in ferrets [24]. Therefore, H9N2 viruses have the potential to adapt to humans, suggesting that once an H9N2 virus acquires such mutations, it could cause a pandemic, since people are immunologically naïve to this HA subtype. However, the molecular basis of H9N2 virus adaptation in humans has not been fully elucidated. Here, we generated mouse-adapted (MA) H9N2 viruses and analyzed the acquired mutations related to mammalian adaptation.

## 2. Materials and Methods

### 2.1. Cells and Viruses

Madin–Darby canine kidney (MDCK) cells, obtained from the American Type Culture Collection (ATCC; CCL-34), were maintained in minimal-essential medium (MEM) supplemented with 5% newborn calf serum (NCS) and antibiotics. Human embryonic kidney 293T cells, obtained from RIKEN BioResource Research Center (RCB2202), and DF-1 chicken fibroblasts, obtained from ATCC (CRL-12203), were maintained in Dulbecco’s modified Eagle’s medium (DMEM) supplemented with 10% and 5% fetal bovine serum (FBS), respectively. All cells were incubated in 5% CO_2_ at 37 °C. A/Hong Kong/1073/99 (H9N2) virus (HK1073) was propagated in 10-day-old embryonated chicken eggs at 37 °C, and the allantoic fluids containing viruses were collected and stored at −80 °C.

### 2.2. Mouse Adaptation of H9N2 Virus

Three 6-week-old female BALB/c mice (Japan SLC, Shizuoka, Japan) were anesthetized with sevoflurane (Maruishi Pharmaceutical, Osaka, Japan) and inoculated intranasally with 50 μL of 10-fold-diluted allantoic fluids containing HK1073 virus in MEM supplemented with 0.3% bovine serum albumin (MEM/BSA). At 3 days post-inoculation, mice were euthanized, and their lungs were collected. The lungs were suspended in phosphate-buffered saline (PBS) containing 2% penicillin and streptomycin and homogenized with a TissueLyser II (QIAGEN, Tokyo, Japan). The homogenates were centrifuged, and the supernatant was collected. Three mice were prepared for the next passage and inoculated intranasally with 50 μL of a 20% lung homogenate. Viruses obtained after 10 passages in mice were utilized as the MA viruses for subsequent experiments.

### 2.3. Sequence Analysis

The RNA of wild-type (HK1073) and MA viruses was extracted from the allantoic fluid of chicken eggs or mouse lung emulsions produced after the tenth passage, respectively. Reverse transcription of viral RNA was performed using primers containing the conserved sequences at the 3′ ends of the viral segments. Then, polymerase chain reaction (PCR) was conducted using specific primer pairs for each gene segment [25]. PCR products were purified using the Fast Gene Gel/PCR Extraction Kit (NIPPON Genetics, Tokyo, Japan) and sequenced directly using specific primers in an automated sequencer (Life Technologies Japan, Applied Biosystems 3170xl, Tokyo, Japan).

### 2.4. Reverse Genetics

The cDNAs of the viral segments obtained from HK1073 or MA viruses were cloned into the genomic RNA expression plasmid pHH21. Escherichia coli DH5α cells transformed with the cDNA-inserted plasmids were incubated at room temperature or 37 °C on LB agar plates supplemented with ampicillin. The coding sequences of the PB2, PB1, PA, and NP segments of the HK1073 and MA viruses were amplified by reverse transcription PCR (RT-PCR) using specific primer pairs and cloned into the protein expression plasmid pCAGGS. All plasmids were sequenced to ensure the absence of unexpected mutations. The primer sequences used in this study will be provided upon request.

Wild-type and mutant HK1703 viruses were generated by reverse genetics as described previously [26]. Briefly, viral RNA expression plasmids and protein expression plasmids were mixed with a transfection reagent (TransIT-293; Mirus Bio, Madison, WI, USA), incubated at room temperature for 15 min, and then mixed with the 293T cells. Transfected cells were incubated in Opti-MEM I (Life Technologies/GIBCO, Grand Island, NY, USA) for 48 h. Supernatants containing infectious viruses were harvested and propagated in MDCK cells in the presence of 1 μg/mL tosylsulfonyl phenylalanyl chloromethyl ketone (TPCK)-trypsin (Worthington, Lakewood, NJ, USA) for 48 h, and then the virus-containing supernatants were aliquoted and stored at −80 °C.

### 2.5. Experimental Infection

Six-week-old female BALB/c mice were anesthetized with sevoflurane and inoculated intranasally with 1 × 10^5^ plaque-forming units (PFU) of wild-type or mutant HK1703 virus in 50 μL of MEM/BSA. The body weights and mortality of the mice were monitored for 14 days. Mice that lost more than 25% of their body weight were euthanized. To assess the replication ability of the mutant viruses in vivo, mice were anesthetized and inoculated intranasally with 1 × 10^5^ PFU of wild-type or mutant HK1703 virus. At 3 days post-inoculation, lungs and nasal turbinates were harvested and homogenized in PBS containing 2% penicillin and streptomycin with a TissueLyser II. Subsequently, the homogenates were centrifuged, and the supernatants were titrated on MDCK cells by plaque assay.

### 2.6. Plaque Assay

Confluent monolayers of MDCK cells were washed with MEM/BSA, infected with diluted virus, and incubated for 60 min at 37 °C. After the virus inoculum was removed, the cells were washed and overlaid with MEM/BSA containing 1% agarose and 1 μg/mL TPCK-trypsin. The plates were incubated at 37 °C for 48 h, and then the virus plaques were counted.

### 2.7. Luciferase Reporter Assay

We prepared plasmids that encode a firefly luciferase gene inserted between the 3’ and 5’ noncoding regions of the NP segment of A/Puerto Rico/8/34 (PR8) under the control of the human PolI promoter (for human 293T cells) or chicken PolI promoter (for chicken DF-1 cells) (named pPolI-NP(0)Fluc(0) and pPolGG-NP(0)Fluc(0), respectively). The pRL-null plasmid (Promega KK, Tokyo, Japan) encodes Renilla luciferase and was utilized as an internal control to normalize transfection efficiency. Human 293T and chicken DF-1 cells were transfected with pPolI-NP(0)Fluc(0) or pPolGG-NP(0)Fluc(0) [27], together with pRL-null and pCAGGS plasmids expressing wild-type PB2, PB1, PA, and NP, or mutant PB1 K577E or PB2 E627K, by using polyethylenimine (PEI; Polysciences, Inc., Warrington, PA, USA). Transfected cells were incubated at 33 °C or 37 °C for 24 h. After incubation, the luciferase activities of the cell lysates were measured and standardized to the activity of Renilla luciferase by using the Dual-Glo Luciferase Assay System (Promega) on an ARVO X2 microplate luminometer (PerkinElmer Japan, Kanagawa, Japan). All experiments were performed in triplicate.

### 2.8. Western Blot Analysis

MDCK cells were infected with viruses at a multiplicity of infection (MOI) of 1 or mock-infected as control. At 12 h post-infection, the cells were lysed on ice for 10 min in lysis buffer (20 mM Tris-HCl, pH 7.6, 100 mM NaCl, 30 mM KCl, 0.1% Triton X-100, and 1 mM EDTA). Deglycosylation of cell lysates was achieved using PNGase F (New England Biolabs Japan, Tokyo, Japan) according to the manufacturer’s instructions. The cell lysates were mixed with 0.05 M dithiothreitol and 2× sodium dodecyl sulphate-polyacrylamide gel electrophoresis (SDS-PAGE) sample buffer (100 mM Tris-HCl, pH 6.8, 4% SDS, 20% glycerol, 25 mM EDTA, and 0.04% bromophenol blue) and boiled for 5 min. Subsequently, the samples were separated by SDS-PAGE and transferred to a polyvinylidene fluoride (PVDF) membrane. The membrane was blocked in 5% skim milk, incubated with anti-HK1073 polyclonal mouse serum obtained by challenging BALB/c mice with HK1073 virus, and then incubated with horseradish peroxidase-linked sheep anti-mouse IgG (GE Healthcare Japan, Tokyo, Japan). The blots were reacted with ECL Prime Western Blotting Detection Reagent (GE Healthcare) and detected with an ImageQuant LAS 4000 mini (GE Healthcare).

### 2.9. Statistical Analysis

Infectivity titers, luciferase activities, body weight changes, or mortalities in virus-infected mice were compared by Student’s *t*-test with two-tailed analysis or the Log-rank test to determine the statistical significance of differences. 

### 2.10. Ethics Statement

Our animal study protocol was conducted at the ABSL2 containment facility in accordance with the Regulations for Animal Care at the University of Tokyo and was approved by the Animal Experiment Committee of the Graduate School of Agricultural and Life Sciences at the University of Tokyo (approval number P17-107).

## 3. Results

### 3.1. Adaptation of H9N2 Influenza Virus to Mice

To explore the molecular basis of mammalian adaptation of H9N2 virus, we intranasally inoculated wild-type A/Hong Kong/1073/99 (HK1073) virus into three BALB/c mice and collected the lungs at 3 days post-infection. The organs were homogenized, inoculated into another BALB/c mouse, and serially passaged 10 times. Then, we obtained three lines of MA H9N2 viruses. After the sixth passage of the virus, the mice in each line showed clinical signs of infection, including decreased activity, huddling, hunched posture, and ruffled fur. We used three virus lines from the tenth passage, referred as HK-MA1, HK-MA2, and HK-MA3, for further analyses.

### 3.2. Sequence Analysis of Mouse-Adapted (MA) Viruses

To identify the amino acid changes in the MA viruses, the complete genomes of the wild-type and HK-MA1, -2, and -3 viruses were sequenced (Table 1). The PB2-E627K and PA-T97I mutations found in HK-MA1 and/or HK-MA2 were previously reported as important signatures for mammalian adaptation in other subtypes of avian influenza viruses [28,29,30,31,32], whereas the PB1-K577E mutation, which had not been previously reported, was only found in HK-MA3, and in the absence of the PB2-E627K mutation. The HA-N132D and N198S/T mutations found in all MA viruses potentially abolished N-glycosylation sites.

### 3.3. Identification of the Mutations Responsible for Mouse Adaptation

**Figure 1:**
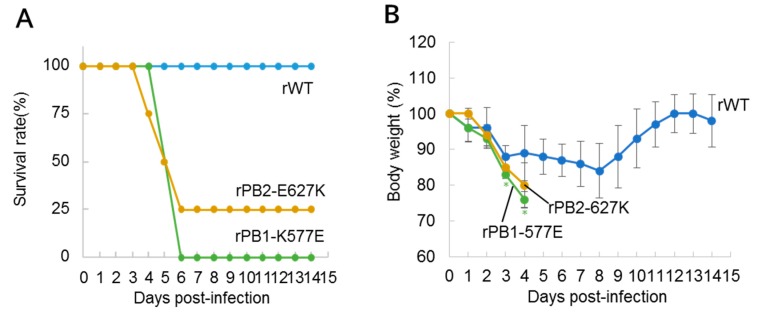

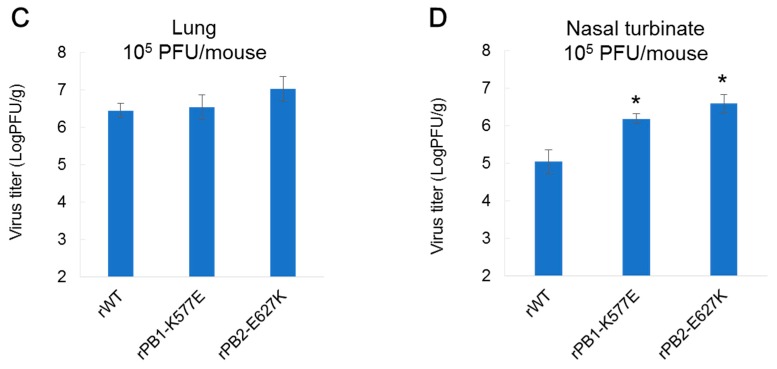
Pathogenicity of polymerase mutant viruses in mice. Six-week-old female BALB/c mice (*n* = 4 per group) were inoculated intranasally with 10^5^ plaque-forming units (PFU) of wild-type (rWT) or mutant (rPB1-K577E or rPB2-E627K) virus per mouse. (**A**) Mortality and (**B**) body weight changes of virus-inoculated mice were assessed for 14 days. Mice that showed a more than 25% body weight loss were euthanized and scored. Significant body weight changes of the mutant virus-infected mice compared with rWT-inoculated mice (*p* < 0.05, Student’s *t*-test, two-tailed analysis) are indicated by an asterisk (*). Replication of rWT, rPB1-K577E, and rPB2-E627K in the (**C**) lungs and (**D**) nasal turbinates of the mice (*n* = 4 per group) at 3 days post-infection. Virus titers (PFU/g) were determined by plaque assay in Madin-Darby canine kidney (MDCK) cells and were presented as mean PFU/g ± standard deviation (SD). Viruses with significant growth enhancement compared the rWT (*p* < 0.05, Student’s *t*-test, two-tailed analysis) are indicated by an asterisk (*).

To identify the mutations responsible for H9N2 virus adaptation to mice, we first generated two recombinant HK1703 viruses, one possessing the PB1-K577E or PB2-E627K mutation, as well as a wild-type virus by reverse genetics (referred as rPB1-K577E, rPB2-E627K, and rWT, respectively). The plaque sizes were similar in all of the three generated viruses in the infected MDCK cells. Then, we intranasally inoculated mice with 10^5^ PFU of each virus and monitored mortality (Figure 1A) and the body weight changes (Figure 1B) for 14 days. All mice inoculated with rWT survived for 14 days, although marked decreases in body weight were observed post-infection. In contrast, those inoculated with rPB1-K577E and rPB2-E627K showed 100% (*p* < 0.01 vs. rWT by Log-rank test) and 75% (*p* < 0.05) mortality, respectively. Only one rPB2-E627K-infected mouse recovered after a 20% body weight loss. To assess the replication properties of the recombinant viruses, we determined the virus titers in the lungs (Figure 1C) and nasal turbinates (Figure 1D) of mice inoculated with 10^5^ PFU of each virus on day 3 post-inoculation. Although the virus titers in the lungs of rWT-, rPB1-K577E-, and rPB2-E627K-inoculated mice were similar, those in the nasal turbinates of mutant virus-inoculated mice were significantly higher than that of rWT-inoculated mice. These data demonstrate that the PB1-K577E mutation was robustly responsible for the increased pathogenicity in mice, as was PB2-E627K mutation.

**Figure 2:**
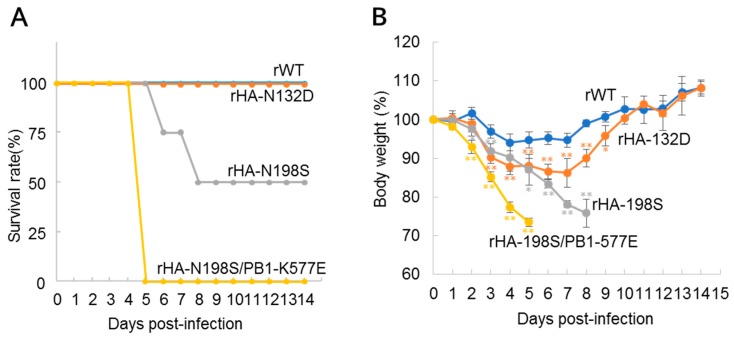
Pathogenicity of hemagglutinin (HA) mutant viruses in mice. Six-week-old female BALB/c mice (*n* = 4 per group) were inoculated intranasally with 10^5^ PFU of rWT, HA mutant (rHA-N132D, rHA-N198S), or double mutant (rHA-N198S/PB1-K577E) virus. (**A**) Mortality and (**B**) body weight changes of the virus-inoculated mice were assessed for 14 days. Mice that showed a more than 25% body weight loss were euthanized and scored. Significant body weight changes of the mutant virus-infected mice compared with rWT-inoculated mice (*p* < 0.05 or *p* < 0.01, Student’s *t*-test, two-tailed analysis) are indicated by an asterisk (*) or two asterisks (**), respectively.

To assess the role of the HA mutations in virus pathogenicity, we next generated two recombinant HK1703 viruses possessing either the HA-N132D or N198S mutation (referred as rHA-N132D or rHA-N198S, respectively). Then, we inoculated mice with 10^5^ PFU of each mutant or rWT virus, which were monitored for 14 days (Figure 2). All mice inoculated with rWT or rHA-N132D survived, although significant body weight losses were observed in the rHA-N132D-inoculated mice compared to those in rWT-inoculated mice. In contrast, half of the mice inoculated with rHA-N198S died by day 8 post-inoculation. Although this reduction in the mortality was not significant owing to a limited number of mice used (Figure 2A), significant body weight losses were observed in the mutant virus-infected mice (Figure 2B). These data demonstrate that, although both HA-N132D and HA-N198S are involved in mouse pathogenicity, the latter had a stronger role than the former.

We also generated a mutant virus containing both HA-N198S and PB1-K577E (referred as rHA-N198S/PB1-K577E), which has the same genotype as HK-MA3, and tested its pathogenicity (Figure 2A,B). All rHA-N198S/PB1-K577E-inoculated mice died by day 5 post-infection, which is a shorter mean death time than that of rPB1-K577E-infected mice (Figure 1A), suggesting a possible synergic effect between these PB1 and HA mutations on pathogenicity.

### 3.4. Viral Polymerase Activity

**Figure 3:**
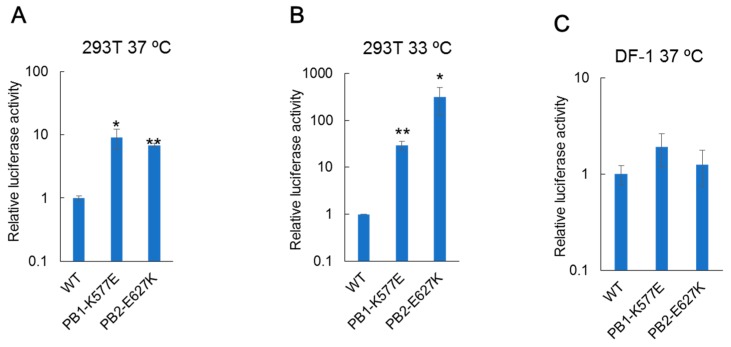
Polymerase activity of PB1-K577E. Polymerase activity was analyzed by a luciferase reporter assay. A plasmid expressing PB1-K577E or PB2-E627K was cotransfected into human 293T or chicken DF-1 cells with four plasmids expressing WT-PB2 (or -PB1), -PA, and -NP plus a reporter plasmid expressing the firefly luciferase gene in a virus minigenome under the control of the human or chicken PolI promoter. WT polymerase activity was also measured as a control. At 24 h post-transfection, cell lysates were subjected to the dual-luciferase assay. Relative polymerase activities are shown as the ratio of firefly luciferase to Renilla luciferase (an internal control) activity and were normalized to WT polymerase levels. Relative polymerase activity in 293T cells at (**A**) 37 °C or (**B**) 33 °C and in (**C**) DF-1 cells at 37 °C. Data are presented as the mean values with standard deviations (SDs) for three independent experiments. Significant increases in polymerase activities compared with that of WT are indicated by a single asterisk (* *p* < 0.05) or double asterisk (** *p* < 0.01).

To examine whether the PB1-K577E mutation affects the polymerase activity of the H9N2 virus, we performed a mini-replicon luciferase assay in mammalian and avian cells at 37 °C and 33 °C, which are the temperatures corresponding to the mammalian lower and upper respiratory tracts, respectively [33]. In human 293T cells at 37 °C, viral polymerase harboring the PB1-K577E mutation showed one log higher activity than WT polymerase, as did the polymerase with the PB2-E627K mutation, which is known to enhance the polymerase activity of avian influenza virus in human cells [28,29,30,31] (Figure 3A). Interestingly, greater enhancement of polymerase activity was observed at 33 °C than at 37 °C; 30-times or 300-times higher activity was observed with PB1-K577E or PB2-E627K mutation, respectively, compared to that of the WT polymerase (Figure 3B). In contrast, there were no significant differences in polymerase activity between WT and mutant polymerases in chicken DF-1 cells at 37 °C (Figure 3C). These data suggest that the glutamate (E) at position 577 of PB1 is important for viral polymerase activity in mammalian cells, leading to the high growth property of the HK-MA3 virus in mouse.

### 3.5. Deglycosylation of Mutant Hemagglutinin (HA) Protein

**Figure 4:**
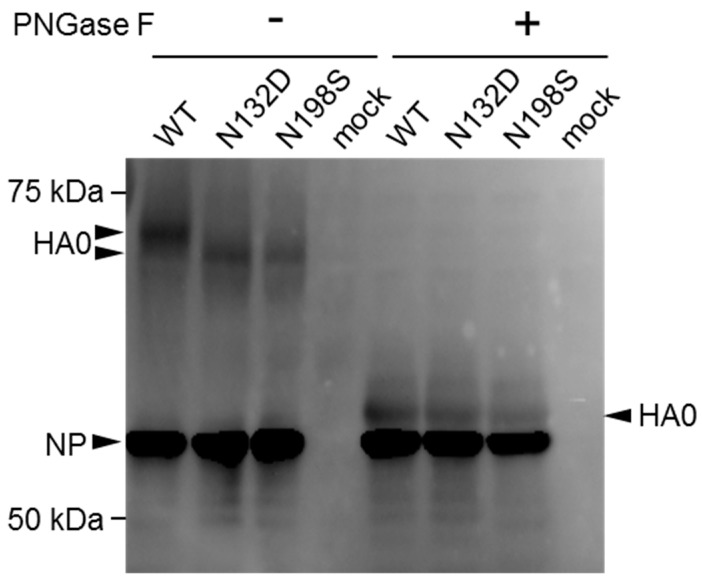
Deglycosylation of HA caused by mutation. MDCK cells were infected with viruses bearing a wild-type or mutant HA (N132D or N198S) and incubated at 37 °C for 12 h. Proteins were extracted from infected or mock-infected cells and treated with or without PNGase F. The samples were run on an 8% sodium dodecyl sulphate (SDS)-polyacrylamide gel and transferred to a polyvinylidene fluoride (PVDF) membrane for Western blotting analysis using anti-H9N2 virus mouse polyclonal antibody as the primary antibody. HA0 and NP are indicated by arrowheads.

We hypothesized that the HA-N132D and HA-N198S/T mutations found in the MA viruses abolished N-linked glycosylation sites. To confirm that these mutations caused deglycosylation of HA, a Western blot analysis was performed. Since the growth of the rHA-N132D and rHA-N198S viruses in cell culture was suboptimal, leading to unclear resolution of the assay, we generated viruses bearing HA-N132D or HA-N198S mutation together with the PB2-E627K mutation by reverse genetics (referred as rHA-N132D/PB2-E627K and rHA-N198S/PB2-E627K, respectively). Then, we inoculated these mutants as well as rPB2-E627K, which possesses wild-type HA, into MDCK cells. After incubation at 37 °C for 12 h, infected cells were lysed and treated with or without PNGase F to remove N-linked glycans. The cell lysates were subjected to Western blot analysis using mouse anti-H9N2 antiserum (Figure 4 and Figure S1). Following PNGase F treatment, HA0 of all samples showed similar mobilities, and were the deglycosylated forms of HA0. In contrast, the mobilities of untreated rHA-N132D and rHA-N198S HA were faster than that of wild-type HA, indicating that they were missing an oligosaccharide side chain. These data suggest that both the HA-N132D and HA-N198S mutations led to the loss of an *N*-glycosylation site.

## 4. Discussion

Worldwide expansion of H9N2 avian influenza virus infection in poultry and sporadic infections in humans are a public health concern, as the virus has the potential to become a pandemic virus. For a new pandemic to emerge, the virus must obtain certain mutations that allow it to adapt to humans. However, the molecular basis of mammalian adaptation of H9N2 virus is not fully understood. Here, we identified a novel PB1 mutation, K577E, in H9N2 viruses serially passaged in mice that could increase pathogenicity in mice, suggesting that the PB1-K577E mutation could be considered to be one of the signatures for mammalian adaptation of avian influenza viruses.

Most avian influenza viruses do not show pathogenicity in mice without initial adaptation. However, the rWT HK1073 virus showed pathogenicity to some extent by inducing a decrease in the body weights upon its infection (Figure 1 and Figure 2). This might be because the HK1073 virus has Ser42 in NS1 and Asp30 and Ala215 in M1, which are known to increase the virulence of H5N1 avian influenza virus in mice [34,35].

Here, we selected H9N2 MA viruses (HK-MA1 and -MA2) harboring the PB2-E627K mutation. Many studies have demonstrated that avian influenza viruses bearing the PB2-E627K mutant show increased replication ability in mammalian hosts and higher virulence in mammals [28,29,30,31]. PB2-E627K is a well-known mutation that increases viral polymerase activity in mammalian cells, and is a critical factor for human adaptation of avian influenza viruses. Separate from this PB2 mutation, we demonstrated that a PB1-K577E mutation increased both the pathogenicity of H9N2 virus in mice (Figure 1 and Figure 2) and polymerase activity in human cells (Figure 3), suggesting that the PB1-K577E mutation, like the PB2-E627K, could contribute to human adaption of avian viruses. In fact, the PB1-K577E mutation enhanced viral polymerase activity in human cells, even at a lower temperature (Figure 3), suggesting the likely acquisition of efficient growth in the upper respiratory tract of humans, an obligatory phenotype of human-adapted viruses.

There was a recent report on the PB1-K577E mutation, showing that this mutation was observed during serial passage of a human H3N2 laboratory strain (A/Hong Kong/1/68) in mice [36]. However, there is no report of the PB1-K577E mutation in regards to mammalian adaptation of avian viruses, such as H9N2 viruses. To investigate the distribution of PB1-K577E mutation in nature, we analyzed the genome sequences of influenza viruses in the NCBI database (https://www.ncbi.nlm.nih.gov/genomes/FLU/Database/nph-select.cgi?go=database) and found out that not only are there no naturally occurring H9N2 isolates possessing a Glu (E) at position 577 of PB1 but also that viruses possessing an E at this position are very rare, even among other subtype mammalian viruses (Table 2). These data imply that an H9N2 virus possessing PB1-577E may not be easily selected in natural settings. However, our finding that the pathogenicity of the H9N2 virus was drastically increased by the PB1-K577E mutation alone provides new insight into the role of the polymerase complex in mammalian adaption of avian influenza viruses.

**Figure 5:**
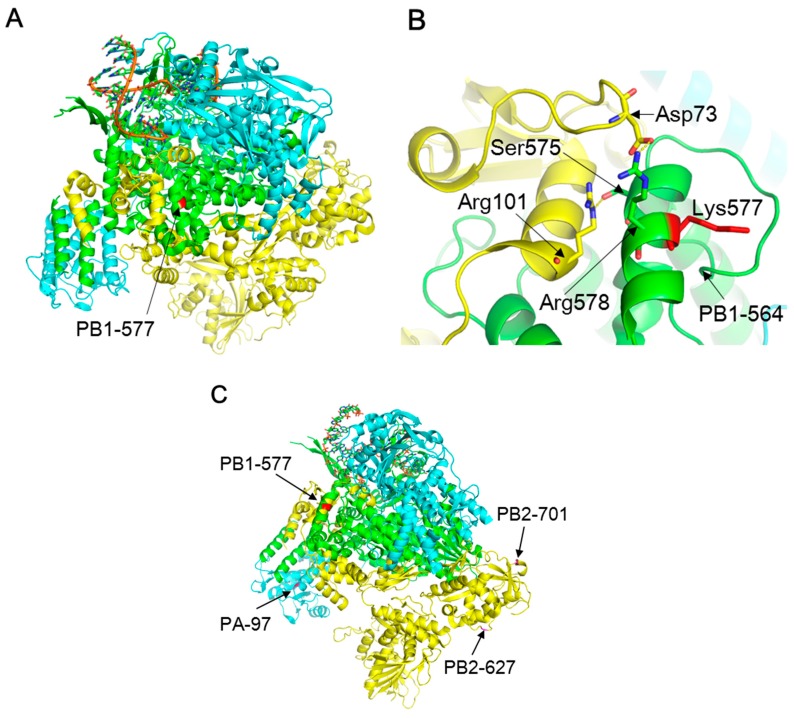
3D structure of the viral polymerase complex (PDB: 4WSB). The PB2, PB1, and PA subunits are shown in yellow, green, and cyan, respectively. (**A**) The amino acids at position 577 in PB1 (PB1-577) are colored in red; (**B**) Close-up view of the vicinity of PB1-577. Polar contacts are made between PB2-Arg101 and PB1-Ser575, and between PB2-Asp73 and PB1-Arg578; (**C**) Amino acid positions PB1-577, PA-97, PB2-627, and PB2-701 are indicated.

The molecular mechanism of the enhanced polymerase activity in mammalian cells due to the PB1-K577E mutation is unknown. A previous study indicated that the PB1 region, comprising residues 506–659, was involved in its interaction with PB2 [37]. The three-dimensional (3D) structure of type A virus polymerase showed that an α-helix formed by residues 573–582 of PB1 made polar contacts with an α-helix formed by PB2 residues 93–102 and 73, and strong interactions were presumed between PB1-575S/PB2-101R and PB1-578R/PB2-73D [38] (Figure 5A,B). Although PB1 residue 577 is not directly involved in these polar contacts, a conformational change in the PB1 α-helix due to the K577E mutation might occur, which could affect the binding affinity for the PB2 α-helix. Since the PB1 subunit of the PB1-PA complex interacts with PB2 in the nucleus [39], the PB1-K577E mutation could alter polymerase activity, likely leading to cold-adaptation of the virus, similar to the PB2-E627K mutation [40].

Another possible mechanism of PB1-K577E-mediated enhancement of polymerase activity is the involvement of a host factor that interacts with the viral polymerase and supports its activity. In the 3D structure, PB1 residues 564–577 appear to form a pocket-like structure, which may form a specific binding site for a host factor. The charge environment in this region may be altered by the K577E mutation due to the change from a negatively-charged K to a positively-charged E, possibly resulting in different binding properties for a host-specific factor. It is well known that the PB2-E627K and PB2-D701N mutations are determinants for mammalian adaptation of avian influenza viruses through their interaction with host species-specific factors, such as importin-α isoforms [41]. Interestingly, the 3D structure revealed that position 577 of PB1 is on the opposite site of positions 627 and 701 of PB2 in the RNA polymerase complex (Figure 5C), suggesting that the host factors which might interact with PB1 may be different from those that interact with PB2. However, the detailed mechanisms including identification of host factors that interact with the PB1 region and structural dynamics of the PB1-K577E mutation need to be analyzed.

A PA-T97I mutation detected in a HK-MA2 virus was previously shown to increase the polymerase activity and mouse pathogenicity of H5N1 avian influenza viruses [42]. In addition, this PA mutation enhanced H6N1 avian virus pathogenicity and expanded tissue tropism in mice when combined with PB2-E627K [32]. This finding resembles those for the mutations found in our HK-MA2 virus. Therefore, PA-T97I is likely important for mammalian adaptation of H9N2 viruses as well.

**Figure 6:**
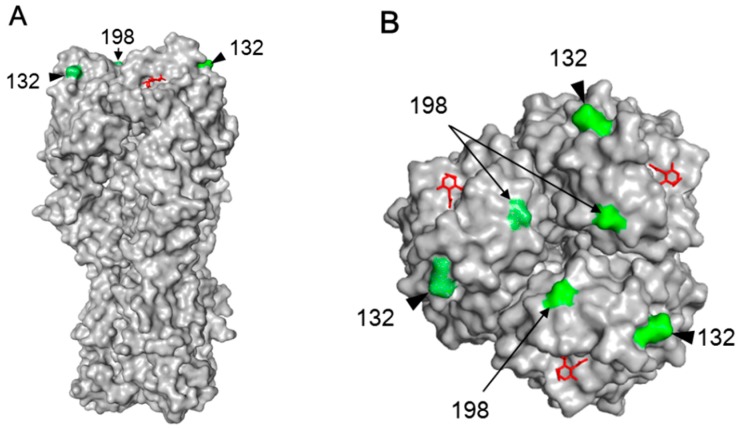
3D structure of HA. Side-view of an HA trimer from H9N2 virus (PDB: 1JSH) (**A**) and its top-view (**B**). Amino acid positions 132 and 198 are colored green. The avian receptor analogue LSTa (LS-tetrasaccharide a: Neu5Ac(α2-3)Gal(β1-3)GlcNAc(β1-3)Gal(β1-4)Glc) is colored red.

The HA-N132D or HA-N198S mutations detected in the HK-MA viruses abolished N-linked glycosylation (Figure 4). Previous reports have shown that H9N2 mutant viruses possessing the HA-N198T mutation, including our HK-MA1 and -MA2 viruses, lack *N*-glycosylation at residue 198 and exhibit higher affinity for the human-type receptor and higher pathogenicity in mice than the parent virus with 198N [43,44]. Our rHA-N198S virus showed increased virulence (Figure 1A,B), possibly due to the same reason. Although there is no previous report of an HA-N132D mutation, the lack of N-glycosylation at residue 132 may alter its receptor-binding property because it is located at a similar distance as the N198 residue to the receptor binding site in the top region of the globular head of HA [45] (Figure 6A,B). However, its receptor binding property may change to a lesser extent compared to the HA-N198S/T mutation, supported by a slight increase in the pathogenicity of rHA-N132D (Figure 2A,B).

In conclusion, a novel PB1-K577E mutation emerges during mouse adaptation of an H9N2 virus, thus we propose PB1-K577E as an additional signature of mammalian adaptation of avian influenza viruses. This finding may contribute not only to the molecular dissection of RNA polymerase but also avian influenza virus surveillance for pandemic risk assessment.

## Figures and Tables

**Table 1:** Mutations detected in mouse-adapted H9N2 viruses.

Virus	Protein	Mutation
HK-MA1	HA	N132D *
		I196M *
		N198T *
	PB2	E627K
HK-MA2	HA	N198T *
	PB2	E627K
	PA	T97I
HK-MA3	HA	N198S *
	PB1	K577E

* H3 numbering.

**Table 2:** Amino acids at position 577 in PB1 of avian, human, and swine influenza virus strains.

Subtype	Avian	Human	Swine
H9N2	K (1277/1283) *	K (13/13)	K (21/22)
	R (6/1283)		Q (1/22)
H1N1	K (475/484)	K (9249/9273)	K (1965/2017)
	R (8/484)	R (16/9273)	R (29/2017)
	E (1/484)	N (6/9273)	N (19/2017)
		Q (1/9273)	T (3/2017)
		M (1/9273)	Q (1/2017)
H3N2	K (304/306)	K (12,138/12,164)	K (1938/1944)
	R (2/306)	R (19/12,164)	R (6/1944)
		Q (2/12,164)	
		T (2/12,164)	
		N (2/12,164)	
		E (1/12,164)	
H5N1	K (1791/1853)	K (183/185)	K (25/26)
	R (61/1853)	R (2/185)	R (1/26)
	E (1/1853)		
H7N9	K (575/576)	K (92/92)	
	E (1/576)		

* Number of strains possessing the indicated residue/total number of strains examined.

## Supplementary Materials

The following is available online at http://www.mdpi.com/1999-4915/10/11/653/s1, Figure S1: Deglycosylation of HA caused by mutaton.
