# Supplementary material for: A PB1-K577E Mutation in H9N2 Influenza Virus Increases Polymerase Activity and Pathogenicity in Mice
Source: Viruses. 2018 Nov 19;10(11):653. doi: 10.3390/v10110653 (PMC6266086; doi:10.3390/v10110653)
Supplement: Supplementary file 1 [file viruses-10-00653-s001.pdf]

Supplementary Information

A PB1-K577E mutation in H9N2 influenza virus increases polymerase activity and pathogenicity in mice

Haruhiko Kamiki, Hiromichi Matsugo, Tomoya Kobayashi, Hiroho Ishida, Akiko Takenaka-Uema, Shin Murakami & Taisuke Horimoto

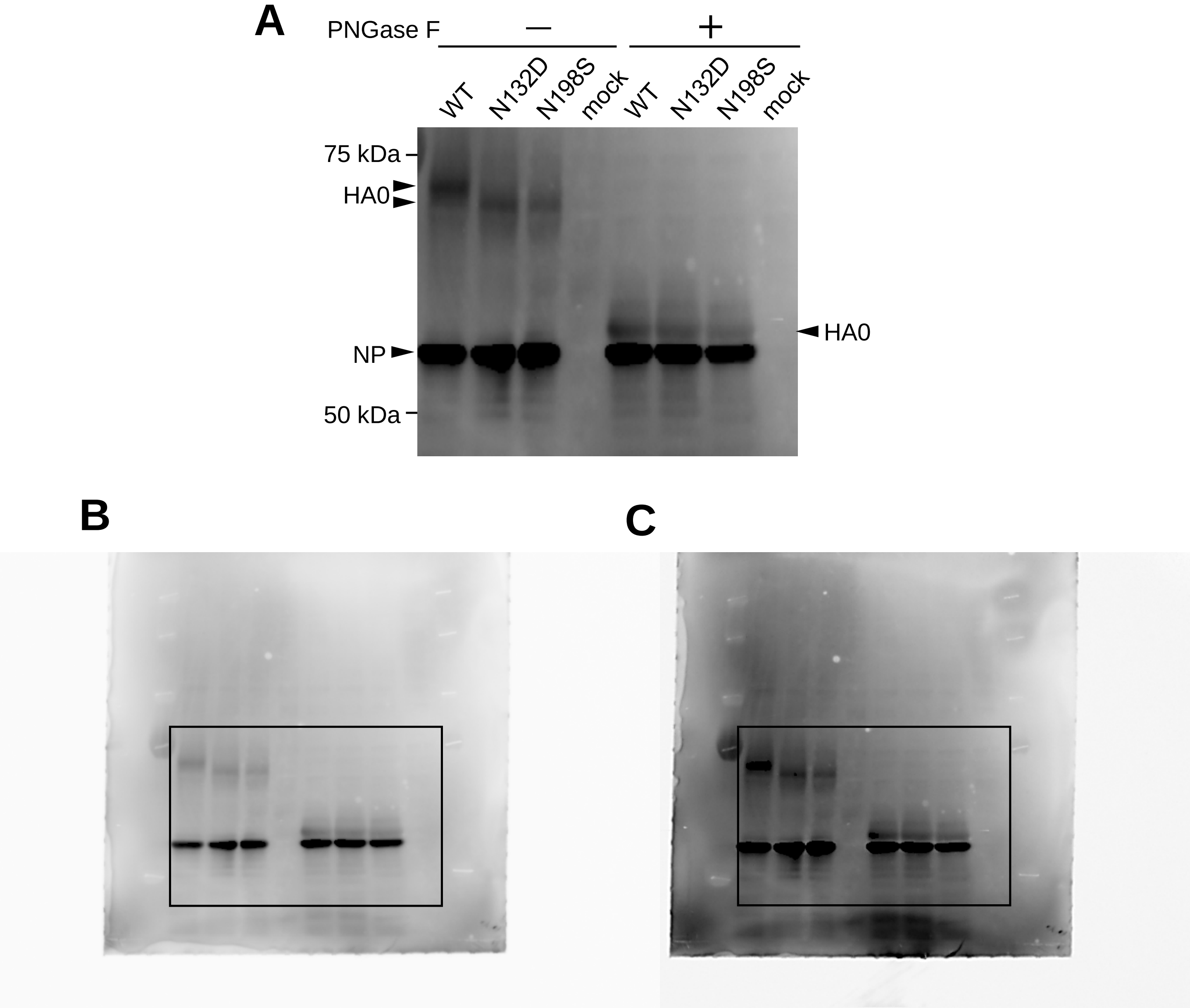

Figure S1. Deglycosylation of HA caused by mutation. MDCK cells were infected with viruses bearing a wild-type or mutant HA (N132D or N198S) and incubated at 37 °C for 12 hours. Proteins were extracted from infected or mock-infected cells and treated with or without PNGase F. The samples were run on an 8% SDS-polyacrylamide gel and transferred to a PVDF membrane for western blotting analysis using anti-H9N2 virus mouse polyclonal antibody as the primary antibody. HA0 and NP are indicated by arrowheads. This gel/blot was uncropped (A). Multiple exposure images of the full-size gels/blots are also shown (B, C).
